# Cognitive function in female patients with chronic functional constipation

**DOI:** 10.1192/j.eurpsy.2022.997

**Published:** 2022-09-01

**Authors:** M. Morozova, G. Rupchev, A. Alexeev, A. Beniashvili, S. Potanin, D. Burminskiy, T. Lepilkina

**Affiliations:** 1 FSBI Research Center of Mental Health, Laboratory Of Psychopharmacology, Moscow, Russian Federation; 2 Moscow State University, Faculty Of Psychology, Moscow, Russian Federation

**Keywords:** chronic functional constipation, female, cognitive function

## Abstract

**Introduction:**

Chronic functional gastro-intestinal disorders can affect cognitive functioning of patients (1). Wong et al (2) showed attentional and executive function impairment, Aizawa et al. (3) found impairment of cognitive plasticity and activity of frontal and temporal arias of brain during performing tests in these patients.

**Objectives:**

Assessing of cognitive functioning of the female patients with chronic functional constipation.

**Methods:**

42 Rome IV adult female patients with chronic functional constipation and 26 adult normative female volunteers were tested with Brief Assessment of Cognition in Schizophrenia (BACS).

**Results:**

Both groups were comparable by age (patients’ group 29,5±6,1, volunteers’ group 28,5±9,6, ns) In both groups subjects demonstrated normal level of cognitive functioning but the scores of the patients were closer to the lower level than the scores of the volunteers. The difference was significant in composite scores and in several other scores (Tab.1). Table1.

**Table:** 

BACS	Patients (n=42)	Volunteers (n=26)	p-level
Verbal Memory	49,5± 10,6	58,8± 7,1	0,0002
Work memory	48,2±10,1	51,7±8,4	ns
Motor token	51,3±10,5	59,8±10,4	0,003
Verbal fluency	52,1±11,5	56,5±9,9	ns
Symbol coding	45,1±8,4	54,8±12,7	0,015
Tower of London (planning)	50,2±10,7	57,6±10,2	0,015
Composite scores	49,1 ±10,1	59,9 ±7,4	0,0001

**Conclusions:**

The findings of the study once more indicate some link between gastrointestinal dysfunction and cognitive functioning. Even these slight decrease in some aspects of cognition from normal population could have negative impact on everyday functioning. The origin of this link is still under question.

**Disclosure:**

No significant relationships.

